# The completeness, accuracy and impact on alerts, of wearable vital signs monitoring in hospitalised patients

**DOI:** 10.1186/s44247-025-00151-x

**Published:** 2025-04-15

**Authors:** Anthony J. Wilson, Alexander J. Parker, Gareth B. Kitchen, Andrew Martin, Lukas Hughes-Noehrer, Mahesh Nirmalan, Niels Peek, Glen P. Martin, Fiona C. Thistlethwaite

**Affiliations:** 1 Division of Informatics, Imaging and Data Sciences, School of Health Sciences, Faculty of Biology, Medicine and Health, University of Manchester, Manchester, UK; 2 Adult Critical Care, Manchester University NHS Foundation Trust, Manchester, UK; 3 Division of Immunology, Immunity to Infection and Respiratory Medicine, School of Biological Sciences, Faculty of Biology, Medicine and Health, University of Manchester, Manchester, UK; 4 Department of Computer Science, School of Engineering, Faculty of Science and Engineering, University of Manchester, Manchester, UK; 5 Division of Medical Education, School of Medical Sciences, Faculty of Biology, Medicine and Health, University of Manchester, Manchester, UK; 6 The Christie NHS Foundation Trust, Manchester, UK; 7 Division of Cancer Sciences, School of Medical Sciences, Faculty of Biology, Medicine and Health, University of Manchester, Manchester, UK

**Keywords:** Wearable sensors, Vital signs monitoring, Early warning scoring systems, Covid-19, SARS-CoV-2

## Abstract

**Background:**

Use of wearable vital signs sensors (WVSSs) to monitor hospitalised patients is growing but uncertainty exists about how such sensors should be adopted into existing practice. The aim of this observational study was to determine the completeness of data capture and accuracy of measurements recorded by a suite of WVSSs. The implications of using such measurements to derive early warning scores was also assessed.

**Methods:**

Adult inpatients with Covid-19 wore four WVSSs recording heart rate/respiratory rate (HR/RR), oxygen saturation (SpO_2_), axillary temperature and blood pressure (BP). Wearable vitals were paired with traditional vitals (measured by nurses) recorded concurrently. The accuracy of the wearable vitals was assessed using traditional vitals as the reference. National early warning (NEWS2) scores were calculated using wearable and traditional vitals.

**Results:**

Forty-eight patients were monitored for 204 days with the sensors. Median sensor wear was 3.9(IQR:1.7–5.9), 3.9(IQR:1.6–5.9) and 3.8(IQR:0.9–5.9) days for HR/RR, temperature and SpO_2_ respectively. The BP cuff was worn for median 1.9(IQR:0.9–3.8) days in 33 patients. Length of hospital stay was 8(IQR:6–13) days. Completeness of data capture was 84% for HR/RR, 98% for temperature, 72% for SpO_2_ and 36% for BP.

There were 1633 HR, 1614 RR, 1412 temperature, 1294 SpO_2_ and 59 BP wearable-traditional measurement pairs. 59.7% of HR pairs were within ± 5 bpm, 38.5% of RR pairs within ± 3breaths/min, 24.4% of temperature pairs within ± 0.3℃, 32.9% of SpO_2_ pairs within ± 2% and 39.0% of BP pairs within ± 10 mmHg. Agreement between wearable and traditional RRs was poor at high RRs.

In a ward setting, 613 NEWS2 scores were calculated using wearable-traditional HR, RR, temperature and SpO_2_ pairs. The median NEWS2_traditional_ was 1(IQR:1–2) and the median NEWS2_wearable_ was 4(IQR:3–6). Using traditional NEWS2 alerts as a reference, 86% (225/262) of wearable NEWS2 5 + alerts and 89% (82/92) of wearable NEWS2 7 + alerts were false positives.

**Conclusions:**

Agreement between vital signs recorded by wearable sensors and concurrent traditional vitals is poor. In this context, data from wearable sensors should not be used in existing track and trigger systems.

**Trial registration:**

The COSMIC-19 study was registered with clinicaltrials.gov (registration: NCT04581031, date of registration: Oct 6th 2020).

**Supplementary Information:**

The online version contains supplementary material available at 10.1186/s44247-025-00151-x.

## Background

Wearable vital signs sensors (WVSSs) are wireless, non-invasive devices worn by patients which permit near-continuous recording of heart rate (HR), respiratory rate (RR), oxygen saturation (SpO_2_) and blood pressure (BP). In low resource settings and healthcare economies struggling with staff shortages, such sensors could automate repetitive manual vital signs measurements thereby freeing time to care. The near-continuous recording theoretically allows no deterioration to go unnoticed and permits advanced analytics to predict and detect important patient centred outcomes and potentially to personalise care. Patients and their relatives may feel a sense of security in knowing that they are always monitored. With these benefits in mind, there has been growing interest [1] in the use of WVSSs to monitor patients in hospital but whether such sensors improve patient outcomes is still to be conclusively established. Recent, single-centre studies have suggested that monitoring with WVSSs can reduce unplanned intensive care unit (ICU) admissions [2] and adverse outcomes [3] but these findings were not replicated in earlier systematic reviews and meta-analyses [4, 5].

Uncertainty concerning the accuracy of the vital signs recorded by WVSSs may partly explain why it has been difficult to establish their impact on clinical outcomes. Numerous studies have assessed the accuracy of vitals recorded by WVSSs but many have focussed on short timescales or have compared WVSSs to ICU standards of monitoring, sometimes in optimised environments [6, 7]. A true picture of their performance requires assessment in hospital wards, ideally following patients through the course of a hospital admission and whilst subject to the challenges and limitations of typical hospital environments.

Most hospitals use some form of track and trigger system to detect and respond to the deteriorating patient [8]. Such systems advocate a protocolised approach to monitoring patients’ vital signs and to alerting when deranged vitals are discovered. In such systems an early warning score (EWS) is often calculated in which patients are assigned a numerical score for each vital sign, with larger scores indicating greater deviation from accepted norms. The EWS is created by summing the component score from each vital with the total used to drive clinical escalation decisions in protocolised track and trigger systems.

Clinical staff do not always follow track and trigger protocols [9] precisely and WVSSs which could automate EWS completion could be valuable. However, studies which assess the impact of incorporating vital signs data from WVSSs into such systems are uncommon [10]. Instead, many authors have suggested developing new approaches to the deteriorating patient which can accommodate data from WVSSs. These have included incorporating alerting from a WVSS to prompt manual calculation of an EWS [11], adjusting EWS systems to reflect wearable data [12, 13] or the development of novel deterioration indices utilising wearable data [14]. A benefit of the EWS system is its simplicity and widespread adoption. New approaches would add complexity. It is therefore prudent to assess what would happen if vital signs from WVSSs were simply used in existing EWS systems.

In this context, we conducted the COSMIC-19 (continuous signs monitoring in Covid-19) study which evaluated a suite of WVSSs in hospitalised patients with Covid-19. We assessed the sensors in a ward environment and endeavoured to continue the assessment even when patients deteriorated. Our primary aim was to determine the completeness and accuracy of the data recorded by the WVSSs in comparison to traditional vital signs. We considered traditional vital signs to be those recorded by healthcare professionals on the ward and in ICU using existing equipment and techniques. Our secondary aim was to assess how National Early Warning Score 2 (NEWS2) scores [15] would differ if calculated using vitals obtained by WVSSs instead of concurrent traditional vitals measurements.

## Methods

The COSMIC-19 study was registered with clinicaltrials.gov (registration: NCT04581031, date of registration: Oct 6th 2020). It was approved by Yorkshire and the Humber – Bradford Leeds Research Ethics Committee, UK (reference 20/YH/0156). It was funded by the Innovate Manchester Advanced Therapy Centre Hub, Innovate UK, (project ID: 6239) with additional support from the Christie Hospital Charitable Fund.

Symptomatic, adult inpatients (16 years or older) with suspected or confirmed Covid-19, who were suitable for escalation to ICU and receiving supplemental oxygen when screened were eligible for enrolment. Patients were recruited within 72 h of hospital admission. We excluded patients unable to give informed consent, who were anticipated to die within 24 h or those with a contraindication to wearing the WVSSs. We recruited a convenience sample of 30–60 participants, informed by the number of WVSSs available for the research. No formal sample size calculations were performed. Some participants were admitted to ICU during the study and monitoring with WVSSs continued unless they were sedated or mechanically ventilated.

Patients were recruited at Manchester Royal Infirmary, an academic, tertiary, metropolitan hospital in the UK with over 150,000 emergency department presentations annually.

### Wearable vital signs sensors

We investigated four WVSSs which measured heart rate/respiratory rate (HR/RR), oxygen saturation (SpO_2_), axillary temperature and systolic blood pressure (SBP). The four sensors were purchased from Isansys Ltd [16] and were used as part of the Isansys Patient Status Engine (PSE), a digital platform for vital signs capture and reporting which is a regulated medical device. Isansys Ltd had no input in the design or conduct of the research. The wearables are summarised in Table 1.

**Table 1:** A summary of the wearable health monitor devices deployed in this study

Feature	Wearable Vital Signs Sensor			
	HR/RR Isansys Lifetouch Sensor [16]	Temperature Isansys Lifetemp Sensor [16]	SpO_2_ Nonin3150 WristOx [17]	BP A&D TM2441 BP Monitor [18]
Form Factor	A small patch attached via standard ECG electrodes	A small adhesive patch	Wrist-worn module, pulse oximetry finger probe	Cuff with attached servo unit.
CE marked medical device	Yes	Yes	Yes	Yes
Wear Location	Precordium	Axilla	Wrist and fingertip	Arm
Method of measurement	HR by single lead ECG. RR derived from HRV using proprietary algorithm.	Thermistor used to determine skin temperature.	SpO_2_ determined from differential absorption spectroscopy.	Automated, oscillometric measurement of blood pressure by sensing cuff.
Frequency of vital sign measurement	Every minute	Every minute	Every minute	Hourly (6am-10 pm), every two hours (10 pm-6am)
Battery Life	72 h	5 days	48 h. 2x AAA batteries.	48 h. 2x AA batteries.
Single Use?	Yes (device replaced when battery low)	Yes (device replaced when battery low)	No	No
Data Synchronisation	BLE to Samsung Galaxy Tablet. Subsequent data upload via WiFi/cellular from tablet to remote server (Isansys LifeGuard Server)			
Software Used	Isansys PSE software			
Metrics recorded	Accelerometer (activity and posture), ECG (single lead), HR, HRV, RR.	Temperature (skin—axilla)	HR (pleth) SpO_2_ (finger)	Non-invasive BP

*BLE* Bluetooth low energy, *BP* blood pressure, *ECG* electrocardiogram, *HR* heart rate, *HRV* heart rate variability, *pleth* plethysmography, *PSE* patient status engine, *RR* respiratory rate, *SpO*_*2*_ oxygen saturation

Participants wore the four WVSSs for up to 20 days or until they were discharged, whichever was earlier. They were free to discontinue any sensor at any time. The research team monitored the participants for WVSS disconnection via a remote dashboard which also allowed researchers to monitor WVSS battery levels. Each participant’s data was reviewed at least once every 24 h. In the event of a disconnection or a low battery, the researchers visited the participant to aid with battery/wearable replacement, wearable re-application or removal if participants chose to discontinue a sensor. The research team maintained a log of all visits and the actions taken during the visits.

### Reference vital signs measurements

Participants received usual care during monitoring with the WVSSs, including traditional vital signs measurements recorded by nurses.

On the ward, according to our institution’s policy, nurses recorded vital signs by direct observation of RR, and measurement of SBP (via arm cuff), SpO_2_ (via finger probe) and HR (via SpO_2_ trace) using the GE Carescape V100 Dinamap vital signs monitor (General Electric Medical Systems Ltd, USA). HR measurements were verified by manual palpation of the radial pulse/cardiac auscultation if required. Temperature was recorded using a Coviden Gen 3 tympanic membrane thermometer (Medtronic Ltd, UK). These traditional vitals measurements were recorded at intervals varying from 1 to 12 hourly in the hospital electronic track and trigger system (Patientrack, Alcidion Ltd, Aus).

In ICU, participants were monitored using a SpaceLabs Xprezzion bedside monitor (SpaceLabs Healthcare Ltd, USA) which continuously recorded HR (via single lead electrocardiogram) and SpO_2_ (via finger probe), and intermittent SBP (via arm cuff). Invasive arterial blood pressure measurements were not included in the analysis. RR and temperature were recorded intermittently by the same methods used on the ward. ICU nurses verified and documented vital signs once every hour from these readings in the ICU electronic patient record (Intellispace Critical Care and Anaesthesia, Philips NV, NL).

### Data capture

An electronic case report form (DataTrial Ltd, UK) was used to capture demographics, diagnostic information, sensor application/removal and outcomes. Traditional vital signs were downloaded from the hospital electronic track and trigger system and the ICU electronic patient record. This was in accordance with local data protection legislation and in accordance with the consent provided by participants. No traditional vital signs measurements were excluded from the analysis except for two HR measurements (0 bpm) and one temperature measurement (5.9℃) which we considered to be data entry errors. Wearable vital signs were captured via the Isansys PSE. Error codes were removed from the wearable data but no vital signs measurements were excluded.

### Data analysis

The completeness of vital sign capture was reported as the percentage of time, in minutes, for which a vital sign was recorded by a WVSS as a proportion of the total time for which the sensor was worn. The number of participants who removed each sensor prematurely and their reasons for doing so was summarised. A Kaplan Meier analysis was conducted where the event of interest was a temporary gap in wearable sensor data of varying durations. Participants were censored if they permanently removed the WVSS for any reason.

For each WVSS, the accuracy of the vital signs was assessed using traditional vital signs as a reference standard. For each traditional measurement, a corresponding wearable vital signs measurement was determined by taking the median of all wearable measurements within the preceding five minutes (for HR, RR, temperature and SpO_2_) or 15 min (for SBP). These timescales and the approach were chosen to align with existing published work [10, 19–24]. A Bland Altman analysis [25] was performed to determine the mean absolute difference (bias) and 95% limits of agreement (LoA) for each vital. The repeated nature of measurements was accounted for using the methods described by Zou et al. [26]. In keeping with previous literature [6], we defined ± 5 bpm, ± 3breaths/min, ± 2%, ± 0.3℃ and ± 10 mmHg a priori as clinically acceptable agreement between HR, RR, SpO_2_, temperature and SBP respectively. The correlation between wearable and traditional measurements was also determined, accounting for the repeated nature of measurements [27].

To assess how monitoring patients with WVSSs would impact clinical alerts, a partial NEWS2 score was calculated using wearable sensor measurements and compared to the NEWS2 calculated from traditional vitals. NEWS2 is the established track and trigger system to assess illness severity and risk of deterioration for hospitalised patients in the UK. This portion of the analysis was limited to vital signs recorded on the ward as EWS are not typically calculated in ICU.

The calculated NEWS2 scores in this study were partial because we did not include level of consciousness and air/oxygen scores as in the full NEWS2 calculation. There are two SpO_2_ scales in NEWS2 with scale 2 reserved for patients with chronic hypercapnic respiratory failure [28]. This did not apply to any participant in this study and we used SpO_2_ scale 1 for all NEWS2 calculations. A modified Clarke Error Grid analysis [29] was also performed for each individual NEWS2 component. This quantifies how differences between corresponding wearable and traditional measurements would impact the NEWS2 component.

All analysis was conducted using R (version 3.6) [30]. Continuous variables were assessed for normality and are presented as mean (standard deviation) or median [interquartile range]. Categorical variables are presented as the population size and the percentage (of available data) for each class.

## Results

### Study participants

**Fig. 1:**
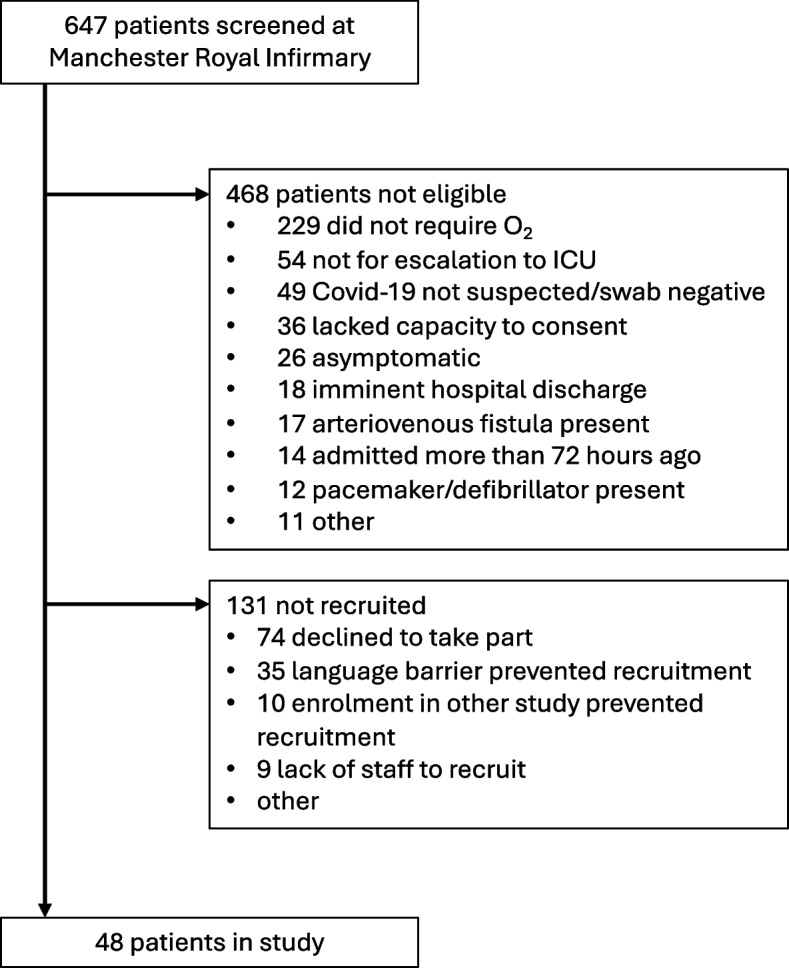
Screening and recruitment to the study

Figure 1 summarises screening and recruitment. 179 eligible patients were identified and 48 took part. Most (43/48) were recruited between July 2020 and March 2021, during the first and second UK waves of the coronavirus pandemic. Five were recruited between July 2021 and February 2022, during the delta and omicron waves in the UK [31]. In 47 participants, SARS-CoV-2 infection was confirmed on nasopharyngeal swab (lateral flow or polymerase chain reaction), one participant had symptoms consistent with Covid-19 and high clinical suspicion of infection but without a positive screening result.

Table 2 summarises the demographics and clinical characteristics of study participants, stratified according to whether they were admitted to ICU. 32/48 (66.7%) were male and 32/48 (66.7%) were from non-Caucasian ethnicities.

**Table 2:** Demographics and clinical characteristics of patients recruited into the study

	**Overall**	**Not admitted to ICU**	**Admitted to ICU**
	***N* = 48**	***n* = 37**	***n* = 11**
Male sex	32 (66.7)	25 (67.6)	7 (63.6)
Age	51.0 [40, 58]	51 [36, 60]	48 [42, 56]
Ethnicity			
Arabic	3 (6.2)	1 (2.7)	2 (18.2)
Indian/Pakistani	20 (41.7)	12 (32.4)	8 (72.7)
Black	8 (16.7)	8 (21.6)	0 (0.0)
Mixed	1 (2.1)	1 (2.7)	0 (0.0)
Caucasian	16 (33.3)	15 (40.5)	1 (9.1)
BMI kg/m^2^ *N* = 43	29.7 [27.0, 34.7]	30.2 [26.7, 34.0]	29.1 [28.2, 37.3]
Type 2 diabetes mellitus	12 (25.0)	8 (21.6)	4 (36.4)
Hypertension	12 (25.0)	8 (21.6)	4 (36.4)
Ischaemic heart disease	2 (4.2)	2 (5.4)	0 (0.0)
CKD (stage 2 +)	4 (8.3)	2 (5.4)	2 (18.2)
Heart failure (NYHA 3 +)	1 (2.1)	1 (2.7)	0 (0.0)
COPD	3 (6.2)	2 (5.4)	1 (9.1)
Asthma	13 (27.1)	9 (24.3)	4 (36.4)
Smoking status			
Current smoker	4 (8.3)	2 (5.4)	2 (18.2)
Ex-smoker	9 (18.8)	7 (18.9)	2 (18.2)
Never smoked	32 (66.7)	25 (67.6)	7 (63.6)
Unknown	3 (6.2)	3 (8.1)	0 (0.0)
Rockwood frailty score			
1	15 (31.2)	11 (29.7)	4 (36.4)
2	17 (35.4)	15 (40.5)	2 (18.2)
3	14 (29.2)	10 (27.0)	4 (36.4)
4	2 (4.2)	1 (2.7)	1 (9.1)

*BMI* body mass index, *CKD* chronic kidney disease classification, *COPD* chronic obstructive pulmonary disease, *ICU* intensive care unit, *NYHA* New York Heart Association classification

### Completeness of data capture from wearable sensors

The median time from admission to wearable sensor application was 27 (IQR:22–46) hours. The median length of hospital stay for each participant was 8 (IQR:6–13) days. The total number of patient-days of sensor wear was 202, 200, 204 and 82 days for the HR/RR, temperature, SpO_2_ and BP WVSSs respectively (Table 3). This represented a median duration of wear of approximately 4 days per participant for the HR/RR, temperature and SpO_2_ sensors. The duration of wear for the BP cuff was median 1.9 days per participant. This was due to connectivity difficulties and because many participants either declined the sensor or requested early removal. The duration of wearable application was similar when calculated from the data recorded by each wearable or from the sensor log maintained by the research team (Appendix 1).

**Table 3:** Duration of wear (first to last valid wearable vital signs measurement), completeness of data capture and reasons for wearable removal. The blood pressure cuff was not applied to 15 participants at their request

	**Wearable Sensor (*N* = 48)**			
	**HR/RR (LifeTouch)**	**Temperature (LifeTemp)**	**SpO**_**2**_** (Nonin PulseOx)**	**BP (A&D TM2441)**
Duration of sensor wear (days/participant)	3.9 [1.7, 5.9]	3.9 [1.6, 5.9]	3.8 [0.9, 5.9]	1.9 [0.9—3.8]
Overall completeness (%) of wearable sensor data	81.2	92.1	68.6	38.4
Completeness (%) per participant	83.8 [64.1, 95.7]	97.7 [79.7, 99.8]	72.3 [61.7, 87.2]	35.8 [16.3, 47.6]
Reason for device removal				
Discharge from hospital	25 (52.1)	23 (47.9)	24 (50.0)	9 (18.8)
Sedated/ventilated on ICU	4 (8.3)	4 (8.3)	3 (6.3)	-
Participant request	19 (39.6)	21 (43.8)	21 (43.8)	22 (45.8)
Never applied	-	-	-	15 (31.3)
Other	-	-	-	2 (4.2)

*BP* blood pressure, *SpO*_*2*_ oxygen saturation

**Fig. 2:**
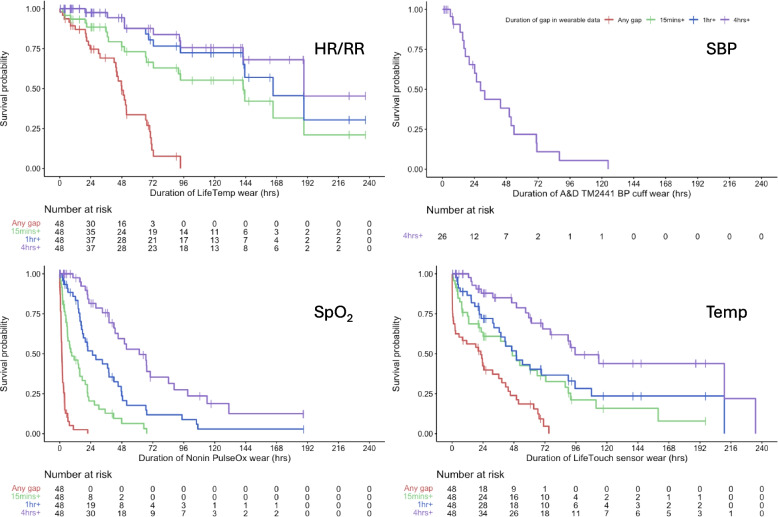
Kaplan Meier analysis of gaps in WVSS data. The event of interest was a temporary gap in wearable sensor data of varying durations. Participants were censored if they permanently removed the WVSS for any reason. HR/RR = heart rate/respiratory rate, SpO_2_ = oxygen saturation, SBP = systolic blood pressure, Temp = temperature

The Kaplan Meier analysis of gaps of varying duration in wearable sensor data is displayed in Fig. 2. The event of interest was the first occurrence, in each participant, of a temporary gap in sensor data of varying durations. The analysis was limited for SBP due to the intermittent nature of measurements (maximum frequency of wearable recordings: 1–2 hourly). Short gaps in data capture were common, but longer, clinically significant gaps in data (4 h or more) were less frequent. Many participants had no such gaps in the first 24 h of wear. Appendix 2 summarises the median survival time without gaps of varying durations and the percentage of patients without such gaps in 24 h. 87.9% of participants had no gaps in HR/RR longer than 4 h in the first 24 h of wear. For SpO_2_, temperature and SBP there were no such gaps in 81.5%, 97.5% and 65.5% respectively.

### Accuracy of wearable sensor measurements

After creating pairs of wearable and traditional vital signs measurements (within the 5/15 min epochs, see “Methods”), there were 1633, 1614, 1412, 1294 and 59 pairs of HR, RR, temperature, SpO_2_ and SBP measurements respectively.

**Fig. 3:**
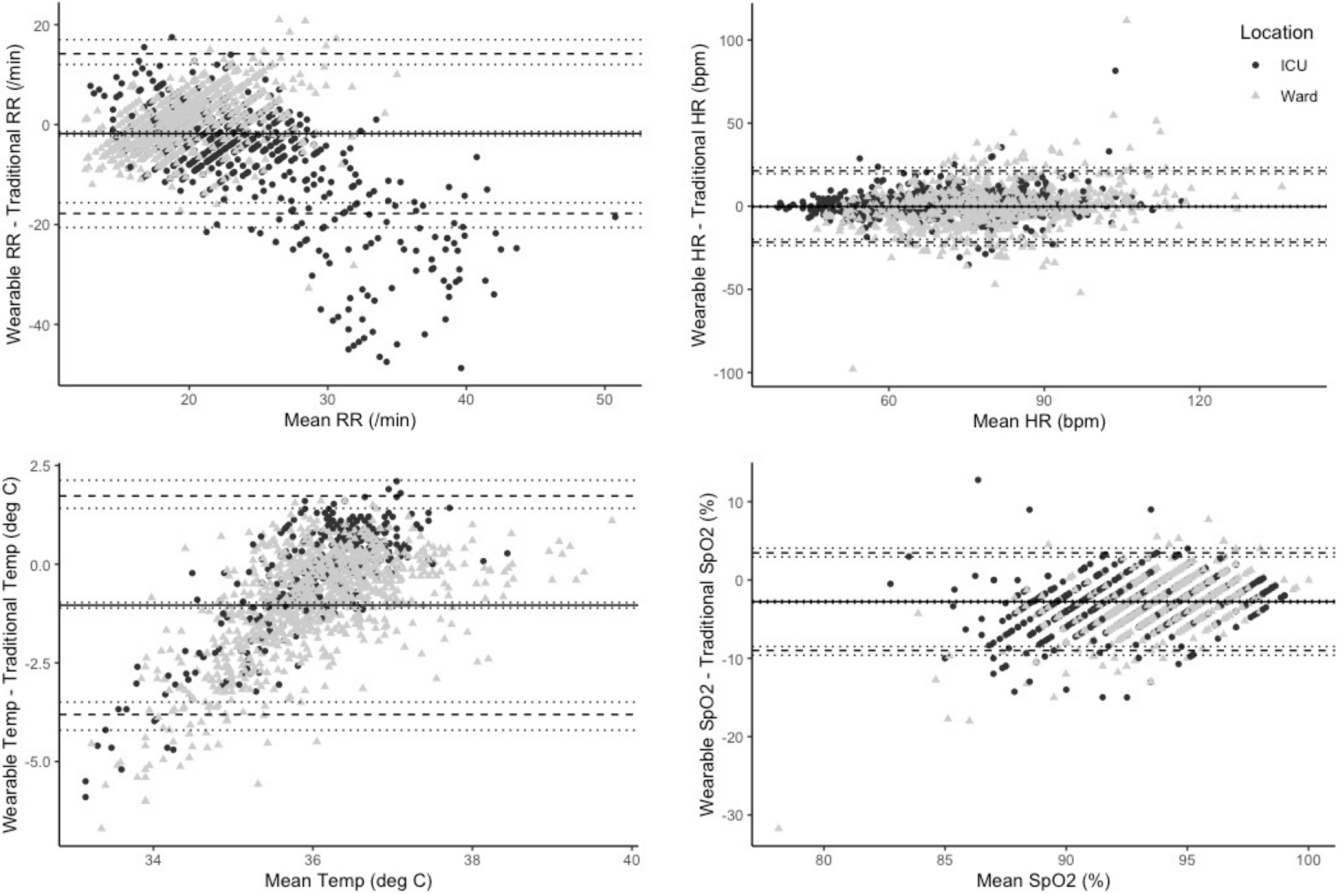
Bland Altman plots corrected for repeated measures. HR = heart rate, RR = respiratory rate, SpO_2_ = oxygen saturation, Temp = temperature. The mean absolute difference (black line) and 95% limits of agreement (dashed lines) plus 95% confidence intervals (dotted lines) are for the entire population of measurement pairs (ward plus ICU measurements)

Figure 3 displays the Bland Altman plots for HR, RR, temperature and SpO_2_ stratified according to whether the participant was on a ward or in ICU. No SBP measurement pairs were recorded in ICU and the Bland Altman plot for SBP is available in appendix 3. Correlation coefficients and associated scatterplots for each vital sign are displayed in appendix 4. There was constant variation between wearable and traditional vital signs measurements across the measurement range for HR and SpO_2_. RR measurements showed a systematic difference at high mean RRs in ICU, with traditional RR measurements being higher than wearable measurements. Temperature measurements also showed a systematic difference at low mean temperatures with traditional measurements being higher than wearable measurements. There were insufficient SBP measurement pairs to comment on the variation in measurements.

Table 4 displays the mean absolute difference (bias) and limits of agreement for each vital sign according to whether the participant was on a ward or in ICU. Not all participants wore all sensors and only participants with at least two measurement pairs are included in the calculations of bias and limits of agreement. Overall, 59.7% HR pairs were within ± 5 bpm, 38.5% of RR pairs were within ± 3breaths/min, 24.4% of temperature pairs were within ± 0.3℃, 32.9% of SpO_2_ pairs were within ± 2% and 39.0% of SBP pairs were within ± 10 mmHg. There was better agreement between WVSS measurements and reference standards for HR measurements on ICU whereas agreement was better for all other vital signs on the ward.

**Table 4:** Bland Altman metrics for each wearable vital sign measurement compared to traditional vital signs measurements recorded on the ward or in ICU as the reference standard

	**Vital signs recorded in a ward setting**				
	**HR (bpm)**	**RR (/min)**	**Temp (℃)**	**SpO**_**2**_** (%)**	**SBP (mmHg)**
Participants^a^	46	46	47	42	10
Measurement pairs	941	940	1049	760	51
Pairs/participant	19 [7,32]	19 [7,32]	20 [8,36]	15 [8,28]	5 [3,5]
Mean absolute difference (bias)	-1.0 (-1.8 to -0.2)	-0.8 (-1.1 to -0.4)	-1.1 (-1.2 to -1.1)	-2.8 (-3.1 to -2.6)	-0.7 (-8.3 to 6.9)
Upper LoA	24.4 (22.0 to 27.2)	11.4 (9.6 to 13.7)	1.5 (1.2 to 1.9)	3.4 (2.8 to 4.1)	37.6 (27.9 to 55.5)
Lower LoA	-26.3 (-29.1 to -23.8)	-12.9 (-15.2 to -11.1)	-3.8 (-4.2 to -3.5)	-9.0 (-9.7 to -8.4)	-39.0 (-56.8 to -29.2)
% within clinically acceptable limits	51.6	42.0	25.6	34.6	39.0
	**Vital signs recorded in an ICU setting**				
	**HR (bpm)**	**RR (/min)**	**Temp (℃)**	**SpO**_**2**_** (%)**	**SBP (mmHg)**
Participants^a^	8	8	8	7	-
Measurement pairs	691	673	362	534	-
Pairs/participant	81 [29,119]	82 [29,114]	47 [18,60]	82 [66,102]	-
Mean absolute difference (bias)	2.2 (1.5 to 2.8)	-7.9 (-8.9 to -7.0)	-0.2 (-0.2 to 0.0)	-2.2 (-2.6 to -1.9)	-
Upper LoA	16.5 (15.3 to 18.2)	16.9 (7.6 to 38.1)	2.5 (1.9 to 3.7)	4.6 (3.0 to 8.1)	-
Lower LoA	-12.2 (-12.8 to -10.9)	-32.7 (-53.9 to -23.5)	-2.9 (-4.1 to -2.3)	-9.1 (-12.5 to -7.5)	-
% within clinically acceptable limits	70.8	33.7	21.0	30.5	-

Mean absolute difference (bias) = wearable—traditional (95% confidence interval), LoA = 95% limits of agreement (95% confidence interval)

*HR* heart rate, *RR* respiratory rate, *SBP* systolic blood pressure, *SpO*_*2*_ oxygen saturation, *Temp* temperature

^a^any participants with only a single measurement pair for the vital sign concerned are excluded from calculations of bias and limits of agreement to enable calculation of the 95% confidence intervals

### Impact of wearable vital signs measurements on alerts

Amongst the pairs of traditional and wearable vital signs measurements, there were only 31 instances in 13 participants when HR, RR, temperature, SpO_2_ and SBP were simultaneously available to calculate a partial NEWS2 (see “Methods”). Appendix 5 summarises the differences in partial NEWS2 scores calculated from these five vital signs and the impact on the rate of NEWS2 5 + or 7 + alerts by each method. This small number of NEWS2 scores reflects that many participants did not wear the BP cuff or chose to remove it early.

In contrast, there were 613 instances in 41 participants when HR, RR, temperature and SpO_2_ were simultaneously available to calculate a partial NEWS2. The median NEWS2 by traditional methods was 1 [IQR: 1–2] and by wearable methods was 4 [IQR: 3–6]. Table 5 summarises the number of times when a NEWS2 5 + or 7 + alert would have been generated by each method. At our institution, NEWS2 5 + would typically alert the ward based medical team, whilst 7 + would typically generate an ICU response.

**Table 5:** Confusion matrix for NEWS2 scores calculated from 4 vital signs (heart rate, respiratory rate, temperature and SpO_2_) using paired traditional and wearable vital signs recorded on the ward. A positive NEWS2 score is considered a score of 5 + or 7 + respectively

		**NEWS2 5 + (4 vitals, traditional)**		**NEWS2 7 + (4 vitals, traditional)**	
		Positive	Negative	Positive	Negative
**NEWS2 5 + (4 vitals, wearable)**	Positive	26 + 11^a^	225		
	Negative	6	345		
**NEWS2 7 + (4 vitals, wearable)**	Positive			5 + 5^b^	82
	Negative			2	519

*NEWS2* national early warning score 2

^a^On 11 occasions, vitals from wearable sensors identified a 5 + NEWS2 event at the same time as traditional measurements but also identified a 5 + NEWS2 event in the preceding 12 h which was not detected by traditional measurements. We considered this to represent early detection of deterioration and therefore a true positive

^b^Similarly, there were 5 instances of early detection of a 7 + NEWS2 event

Appendix 6 displays the differences in NEWS2 component scores for each pair of vital signs measurements. The corresponding modified Clarke Error Grids for each vital sign are displayed in appendix 7. The greatest agreement in NEWS2 component scores was observed for HR (83.3%) and SBP (76.3%). NEWS2 agreement for RR, Temp and SpO_2_ was poor at 52.7%, 43.3% and 28.6% respectively.

## Discussion

In this observational study of hospitalised patients with Covid-19 we monitored 48 individuals using WVSSs during their admission. Our primary aim was to determine the completeness and accuracy of vitals recorded by the WVSSs in comparison to traditional vital signs recorded using standard techniques and equipment on the ward and in ICU. Our secondary aim was to assess how NEWS2 scores on the ward would differ if calculated using vitals obtained by WVSSs instead of concurrent traditional vitals measurements.

We found that wearable patch/wrist-based sensors can achieve comprehensive data capture in an inpatient setting with modest (once daily) intervention to maintain the devices. In contrast, an automated, arm-worn, blood pressure cuff was poorly tolerated and unacceptable to most participants. The accuracy of measurements from the WVSSs varied when compared with ward and ICU reference standards but was generally poor with only HR measurements achieving clinically acceptable agreement for more than 50% of the time. The implication of this poor agreement was illustrated by the NEWS2 scores calculated using wearable vitals which were typically higher than those calculated using traditional vitals. When compared with traditional vital signs, NEWS2 alerts (5 + and 7 +) derived from WVSSs were false positives in most cases.

Studies in which WVSSs have been assessed in ward-based settings and for prolonged periods (> 48 h) have found completeness of data capture by wearable sensors ranging from 76–96% for patch-based, chest HR/RR sensors [32, 33] and from 50–68% for wrist-worn pulse oximeters [34, 35]. Our results are similar, and variation may be attributed to different patient populations and different levels of experience amongst patients, researchers and clinical teams in using and maintaining the sensors. Isolation measures due to Covid-19 in our study, may have also limited opportunities for sensor maintenance. Even the lowest rate of data capture in our study (SpO_2_, 68.6%) equates to continuous vitals for 16 out of every 24 h, far exceeding what could be captured by traditional methods. 50–60% of patients wore the patch and wrist-based sensors until hospital discharge or until they were sedated/ventilated on ICU, suggesting that sensor wear was acceptable to many if not all patients.

Our survival analysis found that there would be no gaps in the data of over 4 h duration for 87.9%, 81.5% and 97.5% of patients using the HR/RR, SpO_2_ and temperature sensors respectively in the first 24 h of wear. As four hours is often the interval between nurse measured vital signs [36] this suggests that the time nursing staff would need to spend troubleshooting/reapplying such devices would be acceptable. In high-risk surgical populations in the Netherlands, Breteler and colleagues [23, 24] found even greater durability of data capture with wearable sensors. Our results extend this finding to a UK setting with medical inpatients who were subject to isolation restrictions.

In contrast to the patch/wrist-based sensors, capture of BP measurements by an automated, arm-worn cuff in our study was poor. Studies of similar BP sensors have found data capture rates of 44–63% [34, 37] and our rates were lower than this due to technical difficulties and patient discomfort. Over 30% of participants refused to wear the cuff from the outset. This limits the conclusions which we can draw about the A&D TM2441 device as a useful wearable monitor but highlights the importance of considering if such devices are acceptable to patients as an automated, wearable monitor. Inflation of a BP cuff can be uncomfortable, and it may be more tolerable when recorded with a nurse present.

We observed wide limits of agreement between traditional and wearable vitals. Other validation studies using the same WVSSs in post-operative surgical patients [32], in those presenting with acute exacerbation of chronic obstructive pulmonary disease [38], and in labouring women [39] have found narrower limits of agreement for all vital signs but have typically monitored patients for a shorter duration of time, often with direct observation by a researcher [21, 40]. In keeping with previous work, we found that a patch-based, chest sensor underestimated high RRs in critically ill patients [6], that an axillary skin temperature sensor underestimated body temperature when compared to a tympanic reference [21] and that unsupervised, wearable SpO_2_ measurements were typically lower than supervised measurements made using similar sensing technology [32, 38].

The reasons for imperfect agreement between wearable vitals and nurse recorded vitals are myriad. It is recognised that vitals recorded by healthcare professionals are impacted by poor measurement technique, value bias in recording and a Hawthorne effect during measurement [41]. Arguably therefore, WVSSs may offer a truer reflection of a patient’s ongoing, non-observed physiological state. However, systematic errors in wearable vital signs measurements may also play an important role. In our study, the temperature differences we observed may be because estimates of core temperature recorded by an axillary skin sensor are different to estimates recorded by tympanic thermometers. Similarly, RRs determined by a wearable sensor based on an algorithm utilising R-R variation may have been subject to systematic error in Covid-19 patients in whom a relative bradyarrhythmia has been observed [42] (potential for error due to Nyqist sampling limit). Finally, movement artefact during SpO_2_ measurement is arguably more common during continuous monitoring than during brief, supervised assessment of SpO_2_ by a nurse. Previous work has demonstrated that movement artefact leads to underestimates of SpO_2_ [43, 44]. Acknowledging these inherent differences, some authors have suggested that WVSSs should not be compared to nurse recorded “spot” vitals [24]. We disagree, because the comparison serves to illustrate the impact that using WVSSs could have on existing patient deterioration alerting mechanisms in hospitals.

In our study, NEWS2 scores derived from HR, RR, temperature and SpO_2_ measurements were higher when calculated from WVSSs than traditional vitals. Differences in RR, SpO_2_ and temperature NEWS2 component scores were responsible for most of the discrepancy. Similarly, Weenk and colleagues found that two WVSS systems studied on surgical and medical wards [10] both returned higher modified early warning scores than traditional nurse recorded vitals. Our findings suggest that vital signs obtained from WVSSs should not directly replace traditional vital signs in track and trigger systems utilising NEWS2 as this would generate unacceptable levels of false alerts for clinical teams. More work is also needed to confirm the validity of wearable vital signs measurements in settings of illness (very high RR), where a patient’s physiology may be quite different from healthy volunteers.

We propose that a different approach is needed to adopt WVSSs into care pathways for the deteriorating patient. This could include scheduled reviews of wearable sensor trends by healthcare providers [45] as opposed to automated alerts, re-development of EWS thresholds for specific use with wearable sensor data [12, 13] or development of novel, broadly applicable deterioration indices using the granular data which wearable sensors provide or some of the additional metrics which they record. Examples include the presence or absence of micro-events [46] within the continuous data, trend information [47] and heart rate variability [48].

We acknowledge several limitations in our work. Firstly, we studied a population with Covid-19, with a preponderance of Black and Asian participants and a high rate of admission to ICU [49]. This may limit the generalisability of our findings to wider populations without respiratory illness, to those who are less unwell and to those with differing skin pigmentation which may alter pulse oximetry readings [50]. Secondly, our clinical teams were blinded to wearable sensor data and not involved in sensor maintenance which may mean that future deployment of WVSSs in broader inpatient groups could achieve better data capture. Thirdly, the variation in number of vital sign pairs analysed for each participant may have introduced bias in our validation results, although we sought to account for this by adjusting for repeated measures throughout our analysis. Finally, the precise time that traditional vital signs were measured was unknown, in that there may be a delay between measurements and recording in the electronic patient record by our nursing staff. Whilst this could impact our validation results, the guidance at our institution is that recording of vital signs should be performed promptly, making it reasonable to assume that measurements were made in the preceding five minutes.

## Conclusion

In hospitalised patients with Covid-19, a suite of patch-based/wrist worn wearable vital signs sensors achieved comprehensive data capture over prolonged periods of inpatient monitoring. However, the validity of the recorded, wearable vital signs was poor when compared to vitals recorded by nurses on the ward and in ICU using standard techniques.

NEWS2 scores calculated using the wearable sensor data would have been higher and would have generated frequent false alerts in this patient population. Novel approaches are therefore needed to utilise wearable sensor data in track and trigger systems that seek to identify and respond to the deteriorating patient.

## Supplementary Information

Supplementary Material 1.

## Data Availability

The COSMIC-19 dataset is available on request subject to appropriate information governance and data sharing agreements. Interested parties should contact the corresponding author via the email address provided.
