# Supplementary material for: The completeness, accuracy and impact on alerts, of wearable vital signs monitoring in hospitalised patients
Source: BMC Digit Health. 2025 Apr 15;3(1):13. doi: 10.1186/s44247-025-00151-x (PMC11997001; doi:10.1186/s44247-025-00151-x)
Supplement: Supplementary file 1 — Supplementary Material 1. [file 44247_2025_151_MOESM1_ESM.pdf]

The completeness, accuracy and impact on alerts, of wearable vital signs monitoring in hospitalised patients

Supplementary Digital Content

Appendix 1	Completeness of wearable sensor data capture from research team log
Appendix 2	Survival analysis for gaps of varying duration in sensor data
Appendix 3	Bland Altman analysis of systolic BP measurement pairs from traditional and wearable vitals
Appendix 4	Correlation plots (wearable vs traditional for each vital sign)
Appendix 5	Differences in partial NEWS2 scores calculated from all five vital signs (traditional vs wearable) and the impact on the rate of NEWS2 5+ or 7+ alerts by each method
Appendix 6	Differences in NEWS2 component scores for paired vital signs measurements (wearable and traditional)
Appendix 7	Modified Clarke Error Grid analysis of differences in early warning score components (NEWS2) derived from wearable and traditional vitals

Appendix 1

The following table summarises completeness of data capture by each wearable sensor according to the duration of wear defined by the researchers (sensor log) and the wearable itself. This serves for comparison with other studies where completeness of data capture is variably defined and provides a check of the robustness of the completeness measurements reported.

A: Completeness from sensor log maintained by research team

Wearable	n	Overall % completeness	Completeness (%, per participant)
HR/RR	48	82.1	86.0 [64.4 – 95.9]
Temp	48	90.7	96.9 [80.7 – 99.3]
SpO_2	48	63.0	62.5 [44.8-74.7]
BP	33	32.9	22.0 [15.0 – 45.0]

B: Completeness from data recorded by each wearable

Wearable	n	Overall % completeness	Completeness (%, per participant)
HR/RR	48	81.2	83.8 [64.1 – 95.7]
Temp	48	92.1	97.7 [79.7 – 99.8]
SpO_2	48	68.6	72.3 [61.7 – 87.2]
BP	30	38.4	35.8 [16.3 – 47.6]

Appendix 1: percentage completeness of data capture for each of the four wearable vital sign sensors worn in the study. The numerator is the total number of minutes for which any vital sign is recorded by the wearable. In A the denominator is the total duration of device wear in minutes determined from the sensor log maintained by the research team. In B the denominator is the total duration of device wear defined as the difference in minutes between the first and last vital sign recorded by the device. HR/RR = heart rate/respiratory rate, Temp = temperature, SpO_2 = oxygen saturations, BP = blood pressure

Appendix 2

Event of interest	Parameter	HR/RR (LifeTouch)	SpO_2 (Nonin PulseOx)	Temp (LifeTemp)	BP (A&D TM2441)
		**Wearable sensor**			
Any data loss	Median survival (hrs)	22.3 (5.4 to 38.5)	1.7 (1.1 to 2.3)	47.9 (43.5 to 67.4)	-
	24hr survival (%)	42.4 (30.2 to 59.4)	0	74.7 (62.7 to 88.9)	-
Data loss >15mins	Median survival (hrs)	46.7 (24.1 to 87.8)	9.3 (6.1 to 19.8)	142 (72.4 to ND)	-
	24hr survival (%)	63.5 (50.7 to 79.7)	20.5 (11.2 to 37.6)	88.4 (79.4 to 98.6)	-
Data loss >1hr	Median survival (hrs)	49.8 (37.9 to 108.0)	25.4 (17.6 to 45.1)	165.2 (142 to ND)	-
	24hr survival (%)	72.0 (59.7 to 86.9)	51.8 (38.3 to 70.2)	97.5 (92.8 to 100)	-
Data loss >4hrs	Median survival (hrs)	95.3 (76.1 to ND)	61.5 (42.9 to 88.5)	189.1 (189.1 to ND)	28.6 (20.1 to 70.7)
	24hr survival (%)	87.9 (78.5 to 98.4)	81.5 (70.0 to 94.9)	97.5 (92.8 to 100)	65.5 (47.7 to 89.9)

Appendix 2: Median and 24-hour survival times until the event of interest (see methods). HR/RR = heart rate/respiratory rate, Temp = temperature, SpO_2 = oxygen saturations, BP = blood pressure

Appendix 3

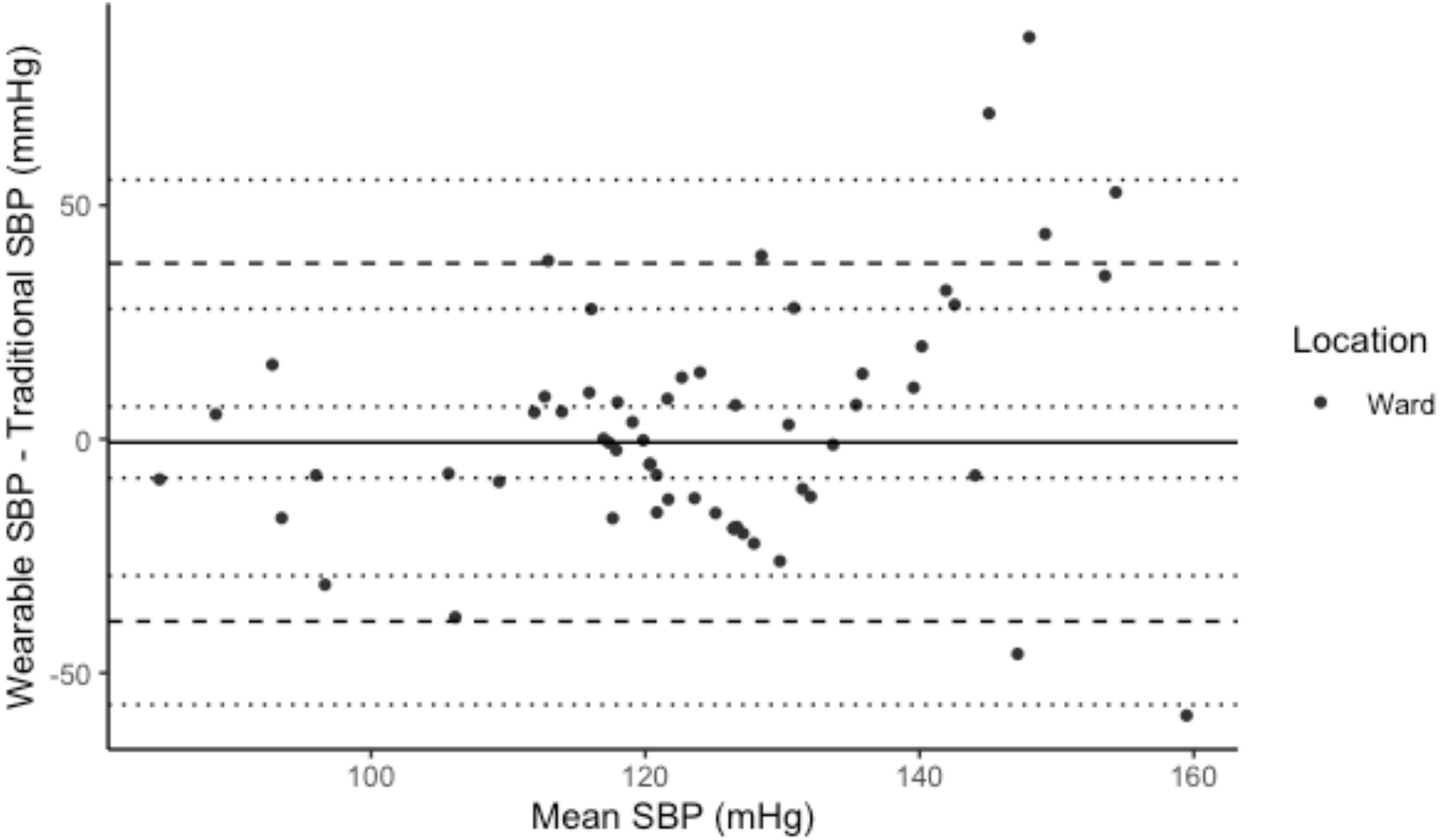

Appendix 3: Bland Altman analysis of systolic BP measurement pairs from traditional and wearable vitals

Appendix 4

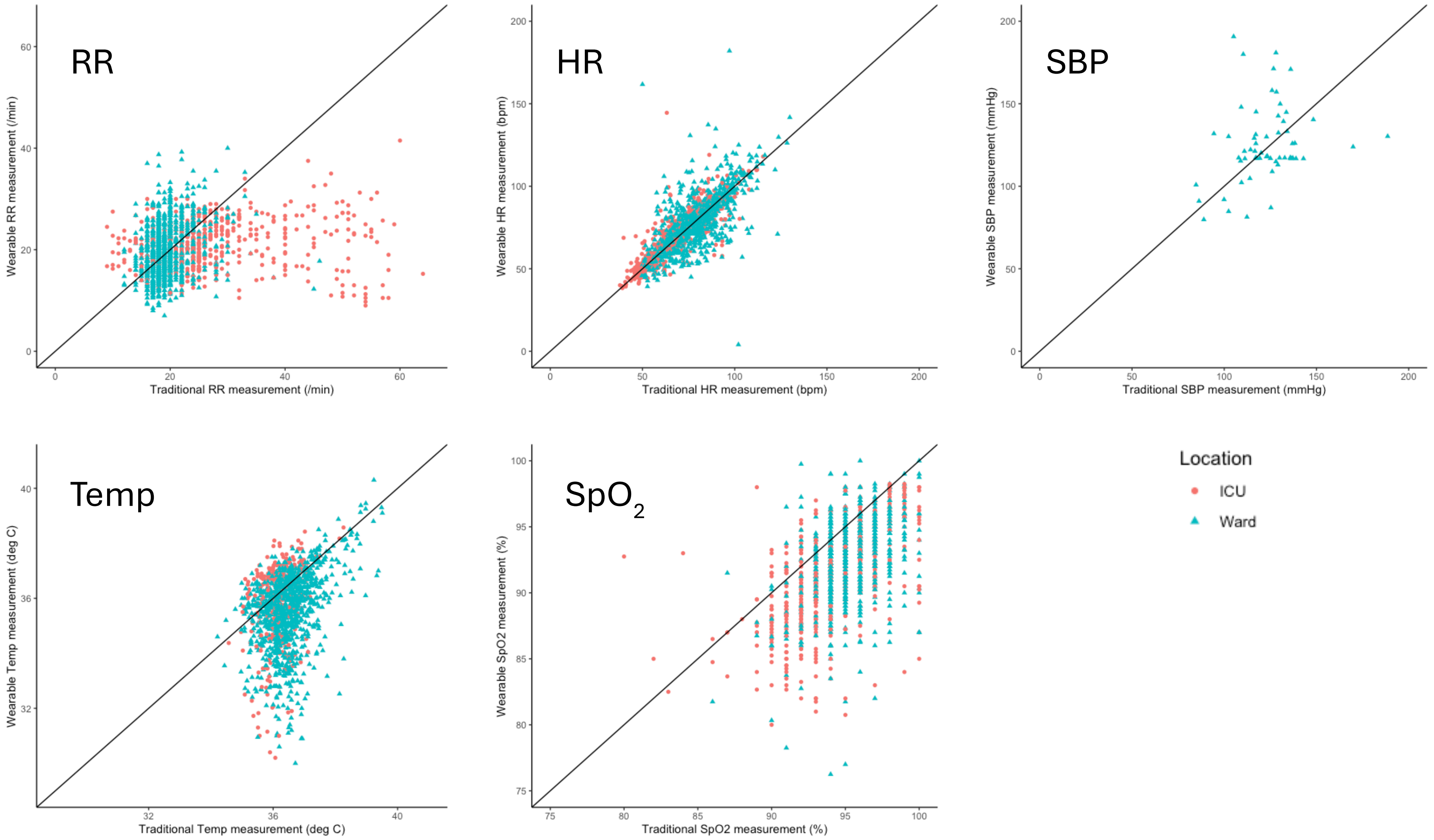

RR
Wearable RR measurement (/min)
Traditional RR measurement (/min)
0
20
40
60
HR
Wearable HR measurement (bpm)
Traditional HR measurement (bpm)
0
50
100
150
200
SBP
Wearable SBP measurement (mmHg)
Traditional SBP measurement (mmHg)
0
50
100
150
200
Temp
Wearable Temp measurement (deg C)
Traditional Temp measurement (deg C)
32
36
40
SpO_2
Wearable SpO2 measurement (%)
Traditional SpO2 measurement (%)
75
80
85
90
95
100
Location
ICU
Ward

	Vital sign				
	**RR**	**HR**	**SBP**	**Temp**	**SpO_2**
Repeated measures correlation coefficient (r_{rm})	0.15 (0.10 to 0.20)	0.63 (0.57 to 0.69)	0.39 (0.17 to 0.57)	0.26 (0.20 to 0.31)	0.36 (0.30 to 0.41)

Appendix 4: Correlation plots between repeated wearable and traditional vital signs measurements. The black line is a line of unity not a line of best fit. HR = heart rate, RR = respiratory rate, Temp = temperature, SpO_2 = oxygen saturations, SBP = systolic blood pressure. The table provides repeated measures correlation coefficients between each traditional and wearable vital sign pair.

Appendix 5

Median NEWS2 (traditional) = 2 [IQR: 1 – 4]
Median NEWS2 (wearable) = 5 [IQR: 3 – 7]

		**NEWS2 5+ (5 vitals, traditional)**		**NEWS2 7+ (5 vitals, traditional)**	
		Positive	Negative	Positive	Negative
**NEWS2 5+ (5 vitals, wearable)**	Positive	5 + 0*	12		
	Negative	2	12		
**NEWS2 7+ (5 vitals, wearable)**	Positive			0 + 0[$]	10
	Negative			21	0

*Appendix 5: confusion matrix for NEWS2 scores calculated from 5 vital signs (heart rate, respiratory rate, temperature, SpO_2 and systolic blood pressure) using paired traditional and wearable vital signs recorded on the ward. A positive NEWS2 score is considered a score of 5+ or 7+ respectively. *On 0 occasions, vitals from wearable sensors identified a 5+ NEWS2 event at the same time as traditional measurements but also identified a 5+ NEWS2 event in the preceding 12 hours which was not detected by traditional measurements. We considered this to represent early detection of deterioration and therefore a true positive. [$]Similarly there were 0 instances of early detection of a 7+ NEWS2 event. NEWS2 = national early warning score 2.*

Appendix 6

	NEWS2 component difference	Vital sign (number of measurement pairs) RR (N=941)	HR (N=942)	SpO_2 (N=760)	Temp (N=1050)	SBP (N=59)
W↓	-3	21 (2.2)	-	1 (0.1)	1 (0.1)	-
	-2	61 (6.5)	1 (0.0)	5 (0.7)	6 (0.6)	2 (3.4)
	-1	13 (1.4)	45 (4.8)	51 (6.7)	60 (5.7)	7 (11.9)
W↔	0	496 (52.7)	785 (83.3)	217 (28.6)	455 (43.3)	45 (76.3)
W↑	1	75 (8.0)	101 (10.7)	251 (33.0)	245 (23.3)	2 (3.4)
	2	159 (16.9)	6 (0.6)	183 (24.1)	78 (7.4)	1 (1.7)
	3	116 (12.3)	4 (0.4)	52 (6.8)	205 (19.5)	2 (3.4)

Appendix 6: differences in the early warning score components generated by pairs of wearable and traditional vital sign measurements recorded on the ward (wearable - traditional). NEWS2 = national early warning score 2, HR = heart rate, RR = respiratory rate, SpO_2 = oxygen saturation, SBP = systolic blood pressure, Temp = temperature, W↓ = wearable returns lower NEWS2 component than traditional measurement, W↑ = wearable returns higher NEWS2 component than traditional measurement, W↔ = wearable and traditional return the same NEWS2 component score. SpO_2 NEWS2 component scale 1 was used to evaluate all SpO_2 measurement pairs.

Appendix 7

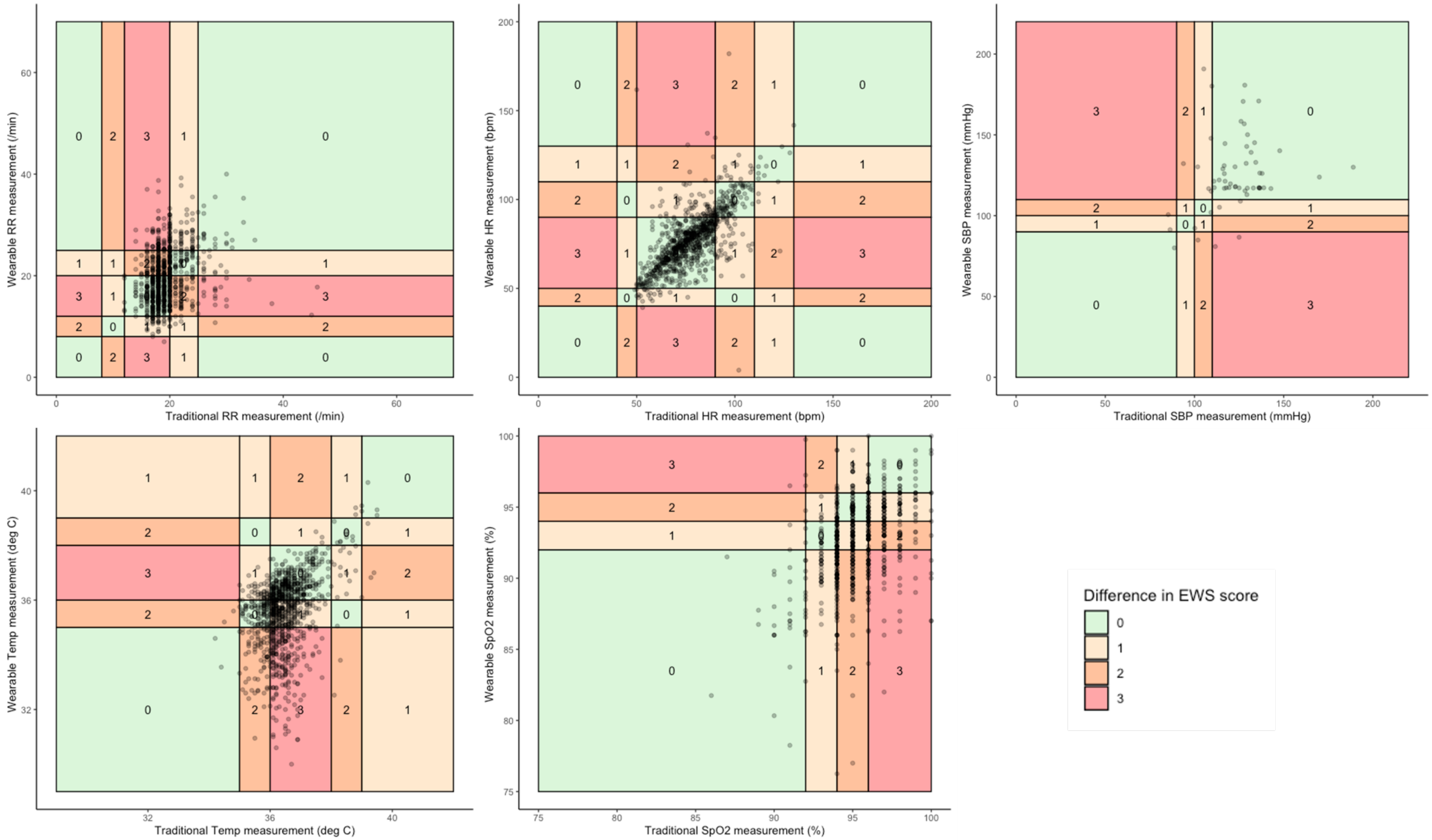

Appendix 7: modified Clark Error grid displaying the difference in NEWS2 component scores for paired wearable and traditional vitals recorded on the ward. NEWS2 = national early warning score 2, HR = heart rate, RR = respiratory rate, SpO_2 = oxygen saturations, SBP = systolic blood pressure, Temp = temperature.
